# Oral health-related quality of life in oral cancer patients assessed with EORTC instruments: a scoping review

**DOI:** 10.1186/s12903-025-07280-9

**Published:** 2025-11-29

**Authors:** You-Bin Yim, Dong-Hun Han, Hye-Sun Shin

**Affiliations:** 1 Department of Preventive and Social Dentistry, School of Dentistry, Seoul National University, 1, Gwanak-ro, Gwanak-gu, Seoul, 08826 Republic of Korea; 2 Dental Research Institute, Seoul National University, Seoul, 08826 Republic of Korea; 3 Department of Dental Hygiene, Dongnam Health University, Suwon-Si, 16328 Gyeonggi- Do Republic of Korea

**Keywords:** Oral cancer, Quality of life, EORTC, Patient-reported outcomes

## Abstract

**Aim:**

This scoping review aimed to map how EORTC instruments have been used to assess oral health-related quality of life (OHRQoL) in patients with oral and oropharyngeal cancer.

**Methods:**

Following JBI and PRISMA-ScR guidelines, a comprehensive literature search was conducted in PubMed, EMBASE, Web of Science, and CINAHL for studies published after 2000. Eligible studies included adult patients with oral/oropharyngeal cancer and reported outcomes using EORTC instruments.

**Results:**

Sixteen studies met the inclusion criteria. Most employed the EORTC QLQ-C30 and H&N35 instruments, assessing OHRQoL across various time points. Results showed that quality of life generally declined after treatment but gradually recovered over time. QoL outcomes varied by tumor stage, HPV status, tumor site, and treatment modality. HPV-positive patients and those treated with surgery alone often reported better functional outcomes. However, EORTC tools lacked sensitivity to capture oral-specific domains such as dry mouth, mouth sores, and sensitivity to food, particularly in advanced-stage patients or those undergoing total glossectomy.

**Conclusion:**

EORTC instruments are widely used in oral cancer research, but may inadequately capture key oral health-specific aspects of patient experience. The EORTC QLQ-OH15 module offers promise in addressing these gaps. Future research should integrate OH15 with existing tools and adopt standardized assessment schedules to enhance comparability and improve patient-centered care.

**Supplementary Information:**

The online version contains supplementary material available at 10.1186/s12903-025-07280-9.

## Introduction

Oral cancer, particularly oral squamous cell carcinoma (OSCC), significantly impacts patients’ quality of life (QoL) due to its effects on speech, mastication, swallowing, and social functioning [1]. Health-related quality of life(HRQoL) generally refers to an individual’s overall perception of physical, psychological, and social well-being influenced by disease and treatment, whereas oral health-related quality of life(OHRQoL) specifically focuses on the functional, psychological, and social consequences of oral health conditions. Given that oral cancer directly compromises essential oral functions and facial appearance, assessing OHRQoL is critical for understanding the unique burden experienced by these patients and for providing a more comprehensive evaluation of treatment outcomes [2, 3].

A variety of QoL assessment instruments have been applied to patients with oral cavity cancer, including general cancer QoL tools—such as the Short Form-36 (SF-36) [4]and the Functional Assessment of Cancer Therapy-General (FACT-G) [5]—head and neck cancer-specific measures like the University of Washington Quality of Life Questionnaire (UW-QoL) [6], and oral health-focused questionnaires including the Oral Health Impact Profile (OHIP) [7]. Each of these tools aims to capture different dimensions of the patient experience. These instruments have provided valuable insights into the multidimensional impact of oral cancer and its treatment on patients’ lives, encompassing functional, psychological, and social domains.

Among the many instruments utilized, those developed by the European Organization for Research and Treatment of Cancer (EORTC) Quality of Life Group have gained particular prominence. The EORTC Quality of Life Group is a leading international research group focusing on developing and coordinating clinical trials to improve cancer treatment and patient outcomes [8]. To evaluate patient well-being, the EORTC Quality of Life Group has developed a comprehensive set of tools to assess QoL in cancer patients [9]. Among these tools, the EORTC QLQ-C30 [8], a core questionnaire addressing general aspects of HRQoL, and its site-specific module for head and neck cancer (H&N35) [10] have been the most widely used instruments for assessing QoL in patients with head and neck malignancies, including oral cancer [11–25].

In this context, a new oral health-specific module, the EORTC QLQ-OH15, was recently developed to improve the assessment of oral symptoms and functional limitations in cancer patients, with emphasis on domains such as dry mouth, sticky saliva, difficulties eating solid food, pain in the mouth, and problems with speech, taste, and mouth opening [26]. Despite its relevance, the QLQ-OH15 has yet to be widely adopted in oral cancer research [27]. Given the emerging complexity of patient-reported outcomes in this field, there is a growing need to examine how existing EORTC instruments have been applied and where the limitations lie in measuring OHRQoL in this population.

Although most previous studies employing the EORTC QLQ-C30 or QLQ-H&N35 were not originally designed to assess OHRQoL as a distinct construct, several subscales of these instruments—such as pain, swallowing, speech, and dry mouth—are closely related to oral health–related domains. Therefore, this review sought to identify how these EORTC instruments have been used to evaluate oral health–related aspects of quality of life among patients with oral and oropharyngeal cancer.

Therefore, this scoping review aimed to (1) map the existing literature regarding the utilization of EORTC questionnaires in assessing OHRQoL in individuals with oral cancer, (2) to ascertain aspects in which OHRQoL varies in studies employing EORTC instruments, and (3) synthesize information related to the practical implementation, perceived utility, and limitations of these tools. Ultimately, this review aims to inform future OHRQoL assessment strategies, including the potential role of more targeted instruments such as the EORTC QLQ-OH15, in oral cancer care and research.

## Methods

### Methodology of scoping review

All articles were processed according to the latest standards recommended by the Johanna Briggs Institute (JBI) [28] and Preferred Reporting Items for Systematic Reviews and Meta-Analyses (PRISMA) Extension for Scoping Reviews (PRISMA-ScR) [29].

### Search strategy

A systematic search of the published literature was conducted in PubMed (NCBI), EMBASE (Elsevier), Web of Science (Clarivate), and the Cumulative Index to Nursing and Allied Health Literature (CINAHL; EBSCOhost), with restrictions to literature published in or after 2000 and limited to studies published in English. The search was conducted on March 4, 2025, based on the following search query.

The search strategy incorporated both controlled vocabulary—Medical Subject Headings (MeSH) and the Emtree thesaurus (Emtree)—and free-text terms. Free-text keywords were specifically searched within the titles and abstracts, and included terms. Boolean operators (“OR”, “AND”) were employed to combine terms and refine the search. The complete search strings for each database are provided in Supplementary file 1.

### Study/source of evidence selection

Following the search, all identified citations will be collated and uploaded to Endnote™ bibliographic management software (Clarivate™, Philadelphia, PA, USA). All results were exported. After duplicates were removed, the study design filter was applied according to the inclusion/exclusion criteria reported in Table 1.

**Table 1:** Inclusion and exclusion criteria

*Inclusion criteria*				
Studies involving patients aged 19 years and older with oral and oropharyngeal cancer				
Studies published in English				
Studies including oral cancer survivors				
Human studies				
Full text peer-reviewed publications				
Studies published after 2000				
*Exclusion criteria*				
Studies involving thyroid or esophageal cancer				
Studies involving nasopharyngeal cancer				
Studies involving metastatic cancer or recurrence				
Studies that do not focus on quality of life outcomes				
Studies involving surgical, dental, economic, or nutritional interventions				
Review articles, meta-analyses, case reports, or conference abstracts				
Studies emphasizing surgical reconstruction or prosthetic rehabilitation rather than EORTC assessment				
Studies focusing on partners or caregivers of oral cancer patients				
Studies in which the analysis or results do not focus on EORTC-based outcomes				

**Fig. 1:**
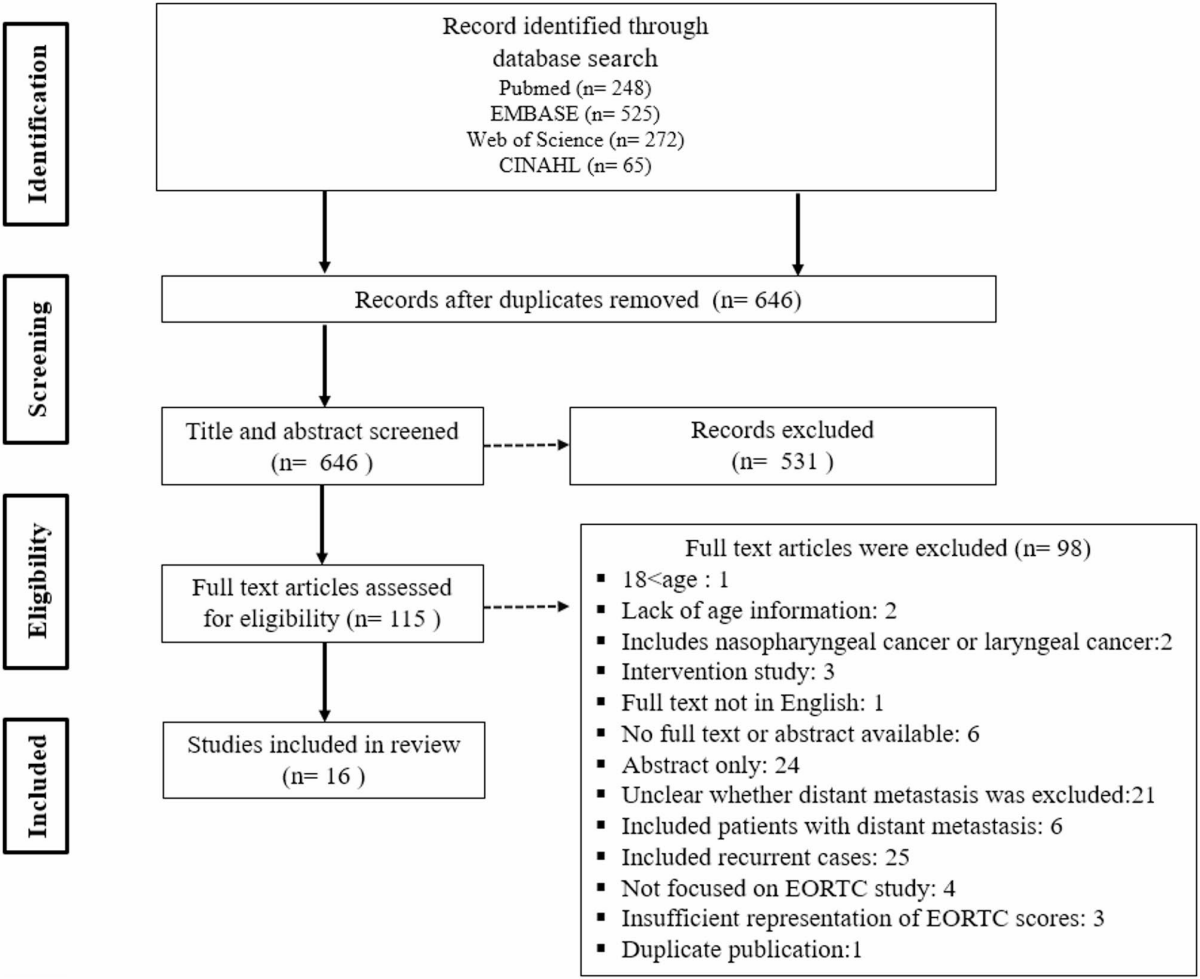
PRISMA search flow diagram

The screening process was conducted in two stages. In the first stage, two authors (H.S.S. and Y.B.Y.) independently screened the titles and abstracts of all retrieved articles to identify potentially relevant studies based on the predefined inclusion criteria. In the second stage, the same two authors independently reviewed the full texts of the selected articles to determine final eligibility. At both stages, any disagreements between the reviewers were resolved through discussion with a third author (D.H.H.), ensuring consensus. The overall screening and selection process is illustrated in the PRISMA flow diagram (Fig. 1).

### Eligibility criteria

Studies were eligible for inclusion if they were original human research articles published in peer-reviewed journals with accessible full texts, written in English, and published after the year 2000. Eligible studies focused on patients aged 19 years or older diagnosed with oral and/or oropharyngeal cancer, particularly survivors, and reported outcomes related to HRQoL. Only analytical observational studies—including prospective and retrospective cohort studies, case-control studies, and cross-sectional studies—were included. In addition, studies were required to include analyses using instruments developed by the EORTC.

Studies were excluded if they involved other cancer types such as thyroid cancer, esophageal cancer, or nasopharyngeal cancer, or if they focused on metastatic or recurrent cancers. Interventional studies, including surgical, dental, economic, or nutritional intervention research, were excluded. Review articles, meta-analyses, case reports, and conference abstracts were also excluded. Furthermore, studies related to reconstructive surgery, prosthetic rehabilitation, or caregivers and partners of oral cancer patients were not considered. Articles that did not perform EORTC-based analyses or did not report results related to EORTC instruments were likewise excluded.

### Data extraction

Data were extracted from the selected studies by two independent reviewers (H.S.S. and Y.B.Y.) using a standardized data collection form in Microsoft Excel (2018 version, Microsoft Corporation; Redmond, WA, USA). The extracted data included the first author, year of publication, journal, study population, sample size (i.e., number of participants), study design, and key outcomes. The dataset was further cross-verified through multiple rounds of review to resolve any discrepancies by consensus. Any disagreements between the reviewers were resolved through discussion, and if consensus could not be reached, a third reviewer (D.H.H.) made the final decision.

## Results

A total of 1,110 records were retrieved from the electronic database. After screening the title and abstract, irrelevant records were removed, leaving the remaining records. After further reading the full text, 16 articles were finally eligible for inclusion and included in this review. The literature screening process is shown in Fig. 1.

### Characteristics of included studies

#### Geographic distribution

The included studies were predominantly conducted in European countries, with three studies from Spain [12, 13, 18], two from Germany [21, 30], and additional studies from the Netherlands [15], Denmark [14], and Italy [11]. Studies from countries such as China [17, 24], the United States [19, 23], France [20], Australia [16], Japan [25], and India [22] were also identified.

#### Study design and population

This scoping review included seven prospective studies [13, 15, 18, 19, 22, 24, 25], 2 retrospective studies [23, 30], and 7 cross-sectional studies [11, 12, 14, 16, 17, 20, 21]. Participant sample sizes within studies varied widely, ranging from 20 to 294. The majority of participants were male, with an age range of 28 to 87.9 years.

#### Tumor site and stage

In all included studies, the tongue was consistently reported as a primary site of oral cancer, with lesions also observed in various locations within the oral cavity and oropharyngeal region. Although some studies excluded stage IV cancers [20, 25], tumor stages ranged broadly from stage I to stage IV. Current therapeutic strategies for oral cavity and oropharyngeal squamous cell carcinoma can be categorized into four primary treatment modalities based on clinical approaches discussed in the review. The primary treatment strategies for oral cavity squamous cell carcinoma (OCSCC) discussed in studies can be categorized into main treatment approaches: surgery, radiotherapy, chemo-radiotherapy, and combination treatments. Detailed characteristics of the included studies, including study design, participant demographics, tumor site and stage, and treatment modality, are summarized in Table 2.

**Table 2:** Study characteristics

First author	Country	Design	Age	Sex	Primary location	Tumor stage	Treatment modality
Baumann et al. 2006 [30]	Germany	Retrospective	41.5–87.9	Total: 34 Male: 24 Female: 10	tonsil, tongue, soft plate	I: 0 II: 8 III: 13 IV: 13	Surgery + radiotherapy: 34
Boscolo-Rizzo et al. 2009 [11]	Italy	cross-sectional	42–77	Total: 57 Male: 48 Female: 9	tonsil, tongue. Soft palate, posterior wall	III: 29 IV: 28	Surgery + radiotherapy: 26 Chemoradiotherapy:31
Infante-Cossio et al. 2009 (1) [13]	spain	prospective	27–83	Total: 69 Male: 51 Female: 18	oral cavity, oropharynx	I, II: 39 III, IV: 29	Surgery: 41 Surgery + chemoradiotherapy: 27
Infante-Cossio et al. 2009 (2) [12]	Spain	cross-sectional	27–83	Total: 128 Male: 95 Female: 33	oral cavity, oropharynx	I, II: 57 III, IV: 71	Not presented
Kjeldsted et al. 2020 [14]	Denmark	cross-sectional	40–90	Total: 179 Male: 128 Female: 51	oral cavity, oropharynx	I, II: 51 III, IV: 128	Surgery: 9 Radiotherapy: 40 Chemoradiotherapy: 109 Surgery and radiotherapy: 18 Surgery + Chemoradiotherapy: 3
Korsten et al. 2021 [15]	Netherland	prospective	59.9(mean)	Total: 270 Male: 179 Female: 91	base of tongue, soft palate, tonsil, orophaynx nos	I: 37 II: 61 III: 59 IV: 113	surgery or radiotherapy: 128 surgery+(chemo)radiotherapy or chemoradiotherapy: 142
McDowell et al. 2021 [16]	Australia	cross-sectional	42–87	Total: 136 Male: 114 Female: 22	tonsil, tongue, pharyngeal wall	I: 74 II: 22 III: 40	Chemotherapy: 120 Radiotherapy: 16
Nemade et al. 2024 [17]	China	cross-sectional	27–74	Total: 25 Male: 24 Female: 1	tongue	III: 7 IV: 18	Surgery: 22 Chemoradiotherapy: 3
Nuñez-Vera et al. 2024 [18]	Spain	prospective	28–85	Total: 72 Male: 61 Female: 11	soft palate, tonsil, tongue, pharyngeal wall	I, II: 30 III, IV: 42	Surgery: 19 Surgery + radiotherapy: 38 Surgery+(chemo)radiotherapy: 6 Chemoradiotherapy: 9
Plonowska-Hirschfeld et al. 2024 [19]	USA	Prospective cohort	41–87	Total: 101 Male: 87 Female: 14	tongue, tonsil, soft palate, glosstonsillar sulcus	T1-3 N0-2	Surgery:42 Surgery + radiotherapy:10 Surgery + chemoradiotherapy:8 Radiotherapy: 11 Chemoradiotherapy:30
Pourel et al. 2002 [20]	France	cross-sectional	41–83	Total: 113 Male: 97 Female: 16	tonsillar fossa, soft palate and uvula, base of tongue, pharyngeal wall	I: 36 II: 42 III: 35	Surgery + radiotherapy: 76 Radiotherapy: 37
Ryzek et al. 2014 [21)	Germany	cross-sectional	36–78	Total: 111 Male: 86 Female: 25	base of tongue, palatine tonsil, palatal arch, oropharynx NOS	T1 = 53 T2 = 58 N0 = 69.2% N1 = 19.2% N2 = 11.5%	Surgery: 26 Surgery + radiotherapy: 34 Surgery + chemoradiotherapy: 51
Sundram et al. 2024 [22]	India	prospective	28–82	Total: 47 Male: 37 Female: 10	tongue	T staging 2: 22 T staging 3: 16 T staging 4 A: 9 N staging 0: 22 N staging 1: 13 N staging 2 A: 5 N staging 2B: 5 N staging 3B: 2	Surgery
Xu et al. 2020 [23]	USA	Retrospective	36–80	Total: 76 Male: 62 Female: 14	tonsil, tongue, pharyngeal wall, softe palate	I: 5 II: 6 III: 14 IV: 46	Surgery:17 Surgery + chemoradiotherapy:23 Chemoradiotherapy:36
Yin et al. 2020 [24]	China	prospective	> 21	Total: 294 Male: 229 Female: 65	base of tongue, tonsil, glossopharyngeal sulcus	T staging 0/1: 124 T staging 2: 117 T staging 3: 53 N staging 0: 120 N staging 1: 125 N staging 2 A/2B: 49	Surgery: 104 Radiography: 131 Combinationa treatment: 59
Yoshimura et al. 2009 [25]	Japan	Prospective	42–88	Total: 20 Male: 15 Female: 5	Tongue, Oral palate, Floor of the mouth, Gingiva, Buccal mucosa, Lip	I: 6 II: 13 III: 1	Radiotherapy

### Quality of life measures

#### Instruments

This analysis demonstrates that all included scoping reviews exclusively incorporated studies utilizing the EORTC QLQ as part of their inclusion criteria. Except for one study [16], all reported results used a combined instrument of the EORTC QLQ-C30 and the EORTC QLQ-HN35 to assess the QoL in patients with oral cancer.

#### Data collection points

Variations in the timing of EORTC instrument administration among studies are likely to reflect differing research aims and methodologies. The EORTC QLQ-C30 and QLQ-H&N35 were used at either single or multiple time points. Single-time assessments occurred at diagnosis before treatment [12], at fixed post-treatment intervals such as ≥ 6 months [23], ≥ 12 months [16], average 18 months post-diagnosis [14], ≥ 18 months post-treatment [21], median follow-up of 2.3 years [30], ≥ 2 years post-initiation [20], ≥ 24 months after treatment [11], and ≥ 5 years post-treatment [17].

Longitudinal designs included two time points (e.g., before and 6 weeks after surgery [22] or before and 3–6 months after treatment [24]; three time points (e.g., before, 1 year, and 3 years after treatment [13, 18]; or at baseline, 3 months, and 1 year post-treatment [19]); four time points (baseline, 3, 6, and 12 months [25]); extended longitudinal follow-up from pretreatment up to 24 months [15].

#### Presentations of quality of life outcomes

Depending on the nature of the collected data, the EORTC scores were reported using different statistical formats. While some studies presented the results as mean values [23, 25] with standard deviations [14, 15, 20, 30] or standard errors [19, 24], others used medians with interquartile ranges [12, 13, 18, 21, 22]. In some cases, the 95% confidence interval of the mean was also reported [11]. A few studies presented all of these statistical measures for each indicator [16, 17]. The key methodological features of the included studies, including the specific EORTC instruments used, timing of data collection, reporting format, and main results, are presented in Table 3.

**Table 3:** EORTC study characteristics

First author	Data collection instrument	Data collection point	Presentation of EORTC Subscale Results	Main results
Baumann et al. 2006 [30]	EORTC QLQ 30 EORTC QLQ HN35	after primary surgery postoperative radiotherapy	• Mean and 95% CI • Comparison: with preoperative EORTC QLQ-C30 and QLQ-H&N35 scores reported by Tschudi et al.(Laryngoscope 2003) and Pourel et al(Int J Radiat Oncol Biol Phys 2002).	• Post-treatment EORTC QLQ-C30 scores in oropharyngeal carcinoma patients showed reduced global health status and role functioning compared to preoperative reference data, with significantly greater appetite loss observed after treatment. • For the EORTC QLQ-H&N35, the most prominent symptoms reported were dry mouth, limited ability to open the mouth, and sticky saliva, and The overall results were largely consistent with previously published reference cohorts
Boscolo-Rizzo et al. 2009 [11]	EORTC QLQ 30 EORTC QLQ HN35	24 monts after treatment	• Mean and 95% CI • Comparison: Surgery + Radiotherapy vs. chemoradiotherapy	• Better long-term QoL was observed in patients undergoing CRT compared to those treated with S + PORT • Surgery had a greater negative impact on physical and social functioning, swallowing, social eating, social contact, fatigue, and pain • Chemo-radiotherapy caused more radiation-related side effects such as dental problems, trismus, dry mouth, and sticky saliva.
Infante-Cossio et al. 2009 [13]	EORTC QLQ 30 EORTC QLQ HN35	before treatment: 1 year after treatment 3 year after treatment	• Median and interquartile range• Comparison: tumor location(oropharynx vs. oral cavity), tumor stage(I + II vs. III + IV), treatment(surgery vs. surgery + radio-chemotherapy)	• QoL gradually improves over time, but certain aspects do not fully return to pre-treatment levels even after three years. • Treatment modality, tumor site, and tumor stage significantly influence quality of life outcomes. • Combined treatment tends to result in poorer QoL compared to surgery alone.
Infante-Cossio et al. 2009 [12]	EORTC QLQ 30 EORTC QLQ HN35	at the time of diagnosis	• Median and interquartile range • Comparison: gender(male vs. female), age (age < 65 vs. age > 65, tumor location(oropharynx vs. oral cavity), tumor stage(I + II vs. III + IV)	• Patients with oral and oropharyngeal cancer often experience significant pre-treatment QoL impairments, especially emotional distress and pain. • Female sex, age under 65, oropharyngeal tumor site, and advanced stage are associated with poorer overall or symptom-specific QoL. • Tumor stage is one of the strongest predictors of QoL at diagnosis.
Kjeldsted et al. 2020 [14]	EORTC QLQ 30 EORTC QLQ HN35	average 18 months after the diagnosis	• Mean and standard deviation • Comparison: HPV negative and HPV positive	• HPV-positive patients tend to have better prognoses, partly due to increased radio sensitivity. • Despite more intensive treatment, HPV-positive OSCC survivors report better outcomes across several HRQL domains. • The relationship between HPV status and HRQL is complex and largely influenced by factors such as age, education, comorbidities, tumor site and stage, treatment modality, smoking, and alcohol use.
Korsten et al. 2021 [15]	EORTC QLQ 30 EORTC QLQ HN35	before treatment: 6 weeks after treatment 6 months after treatment 12 months after treatment 18 months after treatment 24 months after treatment	• Mean and standard deviation presentation • HPV negative and HPV positive	• Among OPSCC patients, the trajectory of HRQOL differs significantly between those with HPV-positive and HPV-negative tumors. • HPV-positive patients tend to have better pre-treatment scores, experience greater short-term deterioration during and after treatment, but show faster and more complete recovery over time. • Global quality of life, physical, emotional, social, role functioning, fatigue, pain, insomnia, appetite loss, and oral pain also follow significantly different patterns between the two groups.
McDowell et al. 2021 [16]	EORTC QLQ 30	at least 12 months after completion of treatment	• Mean, median, standard deviation, interquartile range • comparative Australian nirmal data	• The overall EORTC QLQ-C30 global health status score was comparable to Australian population norms, although survivors reported worse social and cognitive functioning. • Using a two-step clustering analysis of EORTC QLQ-C30 functioning scale scores, the study identified two distinct functioning-based subgroups: a high-functioning group and a low-functioning group. These two subgroups showed large differences on all functioning scales, including physical, role, emotional, cognitive, and social functioning. The low-functioning group reported significantly worse Global Health Status, QoL compared to the HFS, with this difference being interpreted as large
Nemade et al. 2024 [17]	EORTC QLQ 30 EORTC QLQ HN35	5 years after treatment	• Mean, standard deviation, Median, interquartile range	• The EORTC QLQ-C30 showed overall good global health status (M = 80) and good mean scores on the functional scales, ranging from 87 to 91. A mean global health status score of M = 83 was also reported. • The EORTC QLQ-H&N35 indicated that total glossectomy patients experienced problems including pain (31.6), swallowing difficulties (23.3), Speech issues (26.1), and social eating problems (22.4). Single-item problems related to dentition, mouth opening, dry mouth, and cough were also observed.
Nuñez-Vera et al. 2024 [18]	EORTC QLQ 30 EORTC QLQ HN35	before treatment 1 year after treatment 3 year after treatment	• Median and interquartile range • Comparison: tumor stage(early vs. advanced), treatment (surgery/surgery + RT/surgery + RT + CT vs. CT-RT), surgical approach(open surgery vs. transoral)	• Tumor stage and treatment play an important role in the perception of QoL in oropharyngeal cancer (OPC) patients. • Patients with more advanced-stage tumors and treated with definitive chemoradiotherapy (CT-RT) presented the worst outcomes. • Furthermore, patients who underwent open surgery showed significantly greater deterioration in physical and role functioning compared to transoral surgery. • While QoL tends to deteriorate during the first year after treatment, a pattern of stabilization or improvement is observed by the 3-year time point, reaching values similar to or exceeding baseline in most QoL items.
Plonowska-Hirschfeld et al. [19]	EORTC QLQ 30 EORTC QLQ HN35	pretreatment 3 months after treatment 12 months after treatment	• Mean, standard error • Comparison: surgery, surgery + radiotherapy, surgery + CRT, CRT	• EORTC QLQ-C30: While overall QoL was comparable to pretreatment baseline at both 3 months and 1 year across treatment modalities, some groups, particularly S-CRT, reported significant short-term (3-month) declines in functioning, fatigue, and pain which generally improved by 1 year • EORTC HN-35: Significant difficulties with sense of taste and smell persisted at 1 year for all treatment groups, while S-CRT and S-[C]RT patients, compared to SA, also experienced statistically and clinically significant long-term (1-year) issues with salivary dysfunction, social eating, and swallowing, with S-CRT being worst affected.
Pourel et al. 2002 [20]	EORTC QLQ 30 EORTC QLQ HN35	at least 2 years after treatment	• Mean, standard deviation, • Comparison: disease and treatment-related factors (treatment strategy, tumor location, T stage), patient-related factor	• Based on the EORTC QLQ-C30, long-term survivors exhibited significantly impaired QoL, particularly in emotional and social functioning and fatigue, compared to the general population. • The EORTC QLQ-H&N35 results showed prevalent and impaired symptoms such as dry mouth, limited mouth opening, and difficulties with social eating, which were generally unaffected by the initial treatment strategy.
Ryzek et al. 2014 [21]	EORTC QLQ 30 EORTC QLQ HN35	at least 18 months after treatment	• Median, 95% confidence interval of mean • comparison: surgery, surgery + radiotherapy, surgery + che mora diotherapy	• Based on the EORTC questionnaire results for early-stage oropharyngeal carcinoma, the study found that the surgery-only treatment group achieved significantly better outcomes in 11 out of 32 scales compared to groups receiving surgery with radiotherapy or any adjuvant therapy. • These advantages were particularly evident in functional aspects of the head and neck, such as swallowing and speech, but also included improvements in general health and psychosocial factors like pain and financial problems, with no quality of life drawbacks identified for the surgery-only approach.
Sundram et al. 2024 [22]	EORTC QLQ 30 EORTC QLQ HN35	T1: prior to surgery T2: after 6 weeks	• median, interquartile range • comparison: baseline vs. after 6 weeks	• Patients experienced a significant improvement in head and neck cancer-related symptoms six weeks after surgery, particularly in pain, swallowing, and speech-related functions, as reflected by reduced EORTC QLQ-H&N35 scores. • Overall QoL, measured by the EORTC QLQ-C30, remained stable with no significant changes from baseline to the six-week follow-up.
Xu et al. 2020 [23]	EORTC QLQ 30 EORTC QLQ HN35	after completing treatment (mediaan = 2.2 yrs)	• mean • comparison: surgery, definitive (C)XRT, surgery with adjuvant (C)XRT	• For early-stage HPV-associated oropharynx squamous cell carcinoma patients, acceptable QOL is generally achieved regardless of treatment modality, but surgery alone is associated with significantly better outcomes in multiple domains including salivary function, taste, dental, mouth opening, social eating, pain, neck/shoulder, sexual function, and financial issues compared to multimodality treatments. • While patients treated with surgery with adjuvant radiation/chemoradiation (S-a[C]XRT) and definitive radiation/chemoradiation (d[C]XRT) reported generally similar overall QOL, d(C)XRT patients reported significantly worse dental problems, while S-a(C)XRT patients reported significantly worse appearance and coughing.
Yin et al. 2020 [24]	EORTC QLQ 30 EORTC QLQ HN35	before treatment: 3–6 months after treatment	• Mean and standard deviation	• Short-term QOL changes after treatment. Research results indicated that the QoL for patients with HPV-associated oropharyngeal squamous cell carcinoma (OPSCC) generally decreased significantly, and the symptom burden increased, at 3–6 months after definitive treatment compared to before treatment (baseline) • Post-treatment QoL was worse for tumor sites of the tonsil and glossopharyngeal sulcus compared to the base of tongue. Patients with T1 stage disease exhibited relatively better QoL than those with T2 or T3 stage. Furthermore, patients treated with radiation therapy showed better QoL than those treated with surgery or combination therapy
Yoshimura et al. 2009 [25]	EORTC QLQ 30 EORTC QLQ HN35	T1: baseline T2: 3 months after LDR-BT T3: 6 months after LDR-BT T4: 12 months after LDR-BT	• Mean • gender (female vs. male), age (≤ 65 vs. > 65), tumor stage (T stage 1 vs. T-stage 2–3), tumor site (tongue vs. other sites), LDR-BT source (cesium/iridium vs. Au), and complications (with vs. without) on the changes in scores during the first years	• LDR-BT highly maintains QOL in patients with oral cancer. • The changes in QOL during the first year after the start of LDR-BT were unaffected by age, gender, or LDR-BT source, and the changes in only a few functions and symptoms were affected by T-stage, tumor site, and complications.

### Quality of life outcomes

#### Comparison by treatment modality

Treatment outcomes for oropharyngeal carcinoma (OPC) significantly impact patients’ QoL, with variations observed across different therapeutic modalities.

Across most studies, QoL scores tend to worsen significantly after treatment, especially within the first year, but then recover or stabilize within 1 to 3 years, often approaching or even exceeding pre-treatment levels [13, 18]. The EORTC QLQ-H&N35 has shown sensitivity to these longitudinal changes [13]. Treatment modality plays a key role in post-treatment QoL. For advanced oropharyngeal cancer, Boscolo-Rizzo et al. [11] indicate that concurrent chemoradiation therapy (CRT) results in higher overall QoL scores compared to surgery combined with postoperative radiotherapy (PORT). Conversely, Nuñez-Vera et al. [18] suggest that surgery combined with adjuvant radiotherapy/chemotherapy leads to significantly better overall QoL and emotional functioning at 3 years compared to definitive CRT. Despite objective impairments, patients who undergo total glossectomy often report favorable long-term global health-related QoL outcomes, emphasizing the importance of patient-perceived QoL [17].

For early-stage disease, surgery alone showed clear QoL benefits, particularly in role and social function, and fewer symptoms such as nausea, pain, and financial burden [21]. Among HPV-positive OPSCC patients, surgery alone led to better oral function, salivary outcomes, and less xerostomia compared to definitive (chemo)radiation therapy [d(C)RT/d(C)XRT] or surgery with adjuvant (chemo)radiation therapy [S-a(C)RT/S-a(C)XRT] [23].

Although total glossectomy patients reported favorable global QoL outcomes [17], objective assessments revealed significant impairments in speech intelligibility (80–88%) and swallowing (e.g., 84% required special food preparation, 24% had aspiration, and 72% had pharyngeal residue). Long-term symptoms included pain, dysphagia, speech issues, and difficulties in social eating, as well as problems related to dentition, mouth opening, dry mouth, and cough [17].

CRT patients reported more xerostomia and sticky saliva, possibly due to wider radiation fields and concurrent chemotherapy effects [11]. Surgical patients had more issues with swallowing, social eating, and social interaction [11], while CRT patients had worse dental health than those receiving S-a(C)XRT. In turn, S-a(C)XRT patients reported poorer appearance and more coughing than d(C)XRT patients [23].

#### Comparison by HPV status

Across multiple longitudinal studies, patients with human papillomavirus (HPV)–positive oral cancer consistently report higher baseline QoL scores—particularly in domains of physical functioning, social engagement, and overall well-being—compared with HPV-negative patients [14, 18]. During active treatment (surgery, radiotherapy, or concurrent chemoradiation), HPV-positive individuals experience a steeper decline in QoL, reflecting acute side effects such as mucositis, pain, and fatigue [18]. However, from 6 months onward, the HPV-positive group demonstrates a faster trajectory of recovery, often regaining or surpassing their pretreatment QoL levels by 12 to 36 months post-treatment [15, 20]. In contrast, HPV-negative patients show a more gradual improvement, with many domains plateauing below baseline even at long-term follow-up [15].

Although both HPV-positive and HPV-negative groups endure comparable acute toxicities, the long-term domain-specific outcomes diverge significantly. In the role- and social-functioning scales of the EORTC QLQ-C30, HPV-positive survivors score 10–15 points higher on average at 1-year follow-up than HPV-negative survivors, indicating better return to daily activities and social roles [18]. Emotional functioning similarly favors the HPV-positive group by approximately 8 points [14]. On the site-specific QLQ-H&N35 module, HPV-positive patients report 20–25% fewer problems with speech intelligibility and mouth opening, and 15–20% less xerostomia and sticky saliva at 12 months [11]. Taste alteration and oral pain scores also remained 10–12 points lower (better) in the HPV-positive group at both 6- and 12-month assessments [25]. These differences persist—even after adjusting for tumor stage and treatment modality—underscoring the prognostic and functional advantages associated with HPV positivity [18].

#### Comparison by disease stage

Patients with advanced cancer stages (III/IV) generally exhibit lower overall QoL compared to those with early-stage disease [13, 18]. This decline impacts various QoL domains, including pain, fatigue, and issues with swallowing and speech [13, 18].

Oral cancer stage significantly impacts QoL outcomes. Early-stage (T1/T2, I/II) oropharyngeal and oral cavity cancer patients consistently report better overall QoL and functional outcomes, particularly when treated with surgery alone [13, 18, 21, 24]. These patients experience fewer long-term issues with role and social functioning, pain, swallowing, and dry mouth compared to those receiving more aggressive, multimodal treatments [21].

Conversely, advanced-stage (T3/T4, III/IV) patients typically present with and endure significantly worse QoL [13, 17, 18, 22]. They face a higher burden of symptoms including increased fatigue, pain, and marked difficulties in swallowing, speech, and social interaction [12, 17]. Studies on advanced tongue cancer requiring total glossectomy highlight severe impairments in speech understandability and diet consistency, along with moderate to severe issues in objective speech and swallowing evaluations [17]. Furthermore, advanced-stage patients often experience a more severe deterioration in QoL during treatment, with recovery being slower or less complete compared to early-stage counterparts [18]. Treatment intensity, particularly with combination therapies or extensive resections, contributes directly to these poorer QoL outcomes in advanced cases [18, 22].

#### Comparison by tumor location

Tumor location significantly influences QoL outcomes in patients with oral and oropharyngeal cancers. Oropharyngeal tumors generally lead to worse overall QoL and more severe symptoms like fatigue, pain, and difficulties with speech, eating, and social interaction compared to oral cavity cancers [13, 18]. This is often due to the necessity for more aggressive treatments and the greater functional impact on this anatomical region [18].

Within the oropharynx, cancers of the tonsil and glossopharyngeal sulcus may result in worse QoL after treatment compared to tongue cancers [24]. Pharyngeal wall tumors specifically have been associated with poorer functional outcomes in role and social aspects, alongside increased fatigue and pain, and more problems with swallowing, senses, speech, social eating, mouth opening, and dry mouth [20]. For oral cavity cancers, tongue involvement, especially when requiring total glossectomy, leads to significant impairments in objective speech and swallowing, with many patients needing special food preparation or experiencing aspiration [17]. Even when brachytherapy is used for tongue cancers, initial difficulties with swallowing, sensory disturbances, and sticky saliva were more pronounced than in other oral sites, although some symptoms improved over time [25].

## Discussion

This scoping review synthesized findings from studies evaluating QoL or OHRQoL in patients with oral and oropharyngeal cancers. Most studies employed the EORTC instruments, particularly the QLQ-C30 and the site-specific QLQ-H&N35, to assess a broad range of physical, emotional, and functional domains [11, 12, 23]. These modules have been widely validated and remain the cornerstone of QoL assessment in head and neck cancer populations [5]. However, evidence is growing that these tools lack sufficient sensitivity to detect the nuanced burdens associated with oral cavity cancer [6, 8, 9]. In particular, oral function-related symptoms that are especially common and burdensome in oral cancer patients—such as dysphagia, speech difficulties, xerostomia, oral pain, and taste alterations—were only partially addressed and inconsistently reported [11, 14].

Although the QLQ-H&N35 was designed for head and neck cancer populations, it was found to lack sufficient granularity to reflect the unique functional impairments associated with oral cavity cancer, particularly in cases involving total glossectomy, mandibular involvement, or extensive resections [17, 31]. Despite their utility, the heterogeneity in patient-reported outcome measures (PROMs), overlapping content, and variation in interpretation limit comparability across studies [31]. Furthermore, despite their validation, existing tools may not sufficiently capture oral health-specific domains like mastication, sticky saliva, gum discomfort, and mouth discomfort, which are central to the OHRQoL of oral cancer patients [3, 28].

To compensate for these limitations, some studies included additional PROMs, such as the MDADI, OHIP, UW-QOL, and EAT-10, aiming to provide a more granular assessment of functional deficits [16, 22, 25]. These efforts represent valid approaches to obtaining multidimensional perspectives on QoL. However, variability in instruments, overlapping content, and inconsistencies in interpretation limit the comparability and standardization across studies [31]. Despite their widespread validation and utility, these instruments may lack sufficient specificity in addressing oral health-related domains—such as teeth problems, mouth sores, sticky saliva, and oral discomfort—that are particularly relevant to patients with oral cavity cancers [3, 32]. As a result, there is growing recognition of the need for more refined, oral health-focused tools to accurately capture OHRQoL in this patient population [1, 33].

In this context, the recently developed EORTC QLQ-OH15 module presents a promising solution to address these unmet needs. This module includes items that directly target key symptoms frequently reported in the literature, such as dry mouth, sticky saliva, difficulties with solid food, oral pain, speech problems, taste alterations, and restricted mouth opening [34]. These domains are highly relevant to oral cancer patients yet remain underrepresented in existing EORTC tools [34]. Incorporating the OH15 in future studies may enable more sensitive and patient-centered assessments of OHRQoL in this population. Moreover, combining the OH15 with the QLQ-C30 and/or H&N35 could facilitate an integrated approach that captures both general cancer-related and oral-specific QoL issues [34]. This multimodal assessment strategy has the potential to enhance the precision of PROM-based evaluations and ultimately contribute to more individualized survivorship care and clinical decision-making [14, 31].

Furthermore, the wide range of follow-up intervals—from diagnosis/pre-treatment baselines to single post-treatment assessments at 6 months, 12 months, 24 months or even ≥ 5 years—introduces survivorship and recall biases that hinder direct comparisons of domain-specific outcomes across studies. To enhance consistency and comparability between studies, we suggest establishing a more standardized assessment schedule that includes both acute (mid-treatment) and long-term follow-up phases (e.g., baseline, mid-treatment, end of treatment, 6 months, 12 months, 24 months, and ≥ 60 months) to better capture the full trajectory of OHRQoL changes.

Prospective validation of an optimal timing framework for QoL assessments—one that balances patient burden with sensitivity to both treatment-related side effects and long-term functional recovery—will be essential. Future studies should therefore not only integrate more targeted instruments like the OH15 but also adhere to a consensus schedule of time points to enable robust meta-analyses and evidence-based survivorship care planning.

Some strengths and limitations of this scoping review should be acknowledged. This is the first review to systematically map the application of EORTC QoL instruments in patients with oral cancer, with a particular focus on oral health–related domains. However, considerable heterogeneity across the included studies—such as differences in study design, timing of QoL assessments, treatment modalities, and PROMs used—precluded direct comparison of outcomes and limited the ability to conduct a quantitative synthesis. Most studies used different combinations of instruments (e.g., QLQ-C30 with or without H&N35), and reported results using varying formats (e.g., mean scores, medians, or percentages), further complicating data interpretation. Additionally, as is typical of scoping reviews, no formal risk of bias or quality assessment was performed. Lastly, only studies published in English were included, introducing a potential language bias. Therefore, findings should be interpreted as exploratory and hypothesis-generating, rather than confirmatory.

## Conclusion

This scoping review systematically mapped how the EORTC QLQ-C30 and QLQ-H&N35 instruments have been applied to evaluate OHRQoL among patients with oral and oropharyngeal cancers.

1. Through this mapping, we identified how these EORTC questionnaires have been utilized and which OHRQoL domains—such as pain, swallowing, speech, and dry mouth—have been represented.
2. The review also revealed how OHRQoL outcomes varied across studies depending on tumor site, disease stage, HPV status, and treatment modality.
3. Finally, we synthesized findings on the practical implementation, perceived utility, and limitations of these instruments in capturing oral health–specific concerns.

Although the QLQ-C30 and QLQ-H&N35 were originally designed for general and head and neck cancer–specific HRQoL, several of their subscales closely align with oral health–related domains. Building on these insights, the newly developed EORTC QLQ-OH15 presents a valuable opportunity to improve sensitivity and clinical relevance in assessing oral-specific symptoms.

Future studies should integrate the OH15 with existing EORTC instruments, adopt standardized assessment schedules, and extend longitudinal follow-up to better capture both short- and long-term OHRQoL trajectories and inform evidence-based survivorship care.

## Supplementary Information

Supplementary Material 1.

## Data Availability

The datasets generated and/or analyzed during the current study are available from the corresponding author on reasonable request.

## Abbreviations

OSCC: Oral squamous Cell Carcinoma
OPC: Oropharyngeal Carcinoma
EORTC: European Organization for Research and Treatment of Cancer
OHRQoL: Oral Health-Related Quality of Life
HRQoL: Health-Related Quality of Life
JBI: Johanna Briggs Institute
PRISMA-ScR: Preferred Reporting Items for Systematic Reviews and Meta-Analyses (PRISMA) Extension for Scoping Reviews
FACT-G: Functional Assessment of Cancer Therapy-General
UW-QoL: University of Washington Quality of Life Questionnaire
OHIP: Oral Health Impact Profile
EORTC QLQ-C30: European Organization for Research and Treatment of Cancer Quality of Life Questionnaire-Core 30
EORTC QLQ-H&N35: European Organization for Research and Treatment of Cancer Quality of Life Questionnaire– Head & Neck 35
EORTC QLQ-OH15: European Organization for Research and Treatment of Cancer Quality of Life Questionnaire– Oral Health 15
HPV: Human Papillomavirus

## References

[1] Rogers SN, Lowe D. Health-related quality of life after oral cancer treatment: 10-year outcomes. Oral Surg Oral Med Oral Pathol Oral Radiol. 2020;130(2):144–9. 32493685 10.1016/j.oooo.2020.02.018

[2] Mehanna H, Paleri V, West CM, Nutting C. Head and neck cancer–Part 1: epidemiology, presentation, and prevention. BMJ. 2010;341:c4684. 20855405 10.1136/bmj.c4684

[3] Vermaire JA, Partoredjo ASK, de Groot RJ, Brand HS, Speksnijder CM. Mastication in health-related quality of life in patients treated for oral cancer: a systematic review. Eur J Cancer Care (Engl). 2022;31(6):e13744. 36239005 10.1111/ecc.13744 PMC9787816

[4] Herce-Lopez J, Rollon-Mayordomo A, Lozano-Rosado R, Infante-Cossio P, Salazar-Fernandez CI. Assessment of quality of life of oral cancer survivors compared with Spanish population norms. Int J Oral Maxillofac Surg. 2013;42(4):446–52. 23245700 10.1016/j.ijom.2012.11.014

[5] Lin CR, Hung TM, Shen EY, Cheng AJ, Chang PH, Huang SF, et al. Impacts of employment status, partnership, cancer type, and surgical treatment on health-related quality of life in irradiated head and neck cancer survivors. Cancers (Basel). 2024;16:19. 10.3390/cancers16193366 PMC11475803 39409986

[6] Zhu J, Xiao Y, Liu F, Wang J, Yang W, Xie W. Measures of health-related quality of life and socio-cultural aspects in young patients who after mandible primary reconstruction with free fibula flap. World J Surg Oncol. 2013;11:250. 24083617 10.1186/1477-7819-11-250 PMC3850790

[7] Koric A, Chang CP, Hu S, Snyder J, Deshmukh VG, Newman MG, et al. Oral health-related quality of life among oropharyngeal cancer survivors. Oral Oncol. 2024;159:107062. 39362027 10.1016/j.oraloncology.2024.107062 PMC11722133

[8] Aaronson NK, Ahmedzai S, Bergman B, Bullinger M, Cull A, Duez NJ, et al. The European organization for research and treatment of cancer QLQ-C30: a quality-of-life instrument for use in international clinical trials in oncology. J Natl Cancer Inst. 1993;85(5):365–76. 8433390 10.1093/jnci/85.5.365

[9] Bjordal K, Hammerlid E, Ahlner-Elmqvist M, de Graeff A, Boysen M, Evensen JF, et al. Quality of life in head and neck cancer patients: validation of the European organization for research and treatment of cancer quality of life Questionnaire-H&N35. J Clin Oncol. 1999;17(3):1008–19. 10071296 10.1200/JCO.1999.17.3.1008

[10] Bjordal K, Ahlner-Elmqvist M, Tollesson E, Jensen AB, Razavi D, Maher EJ, et al. Development of a European organization for research and treatment of cancer (EORTC) questionnaire module to be used in quality of life assessments in head and neck cancer patients. EORTC quality of life study group. Acta Oncol. 1994;33(8):879–85. 7818919 10.3109/02841869409098450

[11] Boscolo-Rizzo P, Stellin M, Fuson R, Marchiori C, Gava A, Da Mosto MC. Long-term quality of life after treatment for locally advanced oropharyngeal carcinoma: surgery and postoperative radiotherapy versus concurrent chemoradiation. Oral Oncol. 2009;45(11):953–7. 19665919 10.1016/j.oraloncology.2009.06.005

[12] Infante-Cossio P, Torres-Carranza E, Cayuela A, Gutierrez-Perez JL, Gili-Miner M. Quality of life in patients with oral and oropharyngeal cancer. Int J Oral Maxillofac Surg. 2009;38(3):250–5. 19135864 10.1016/j.ijom.2008.12.001

[13] Infante-Cossio P, Torres-Carranza E, Cayuela A, Hens-Aumente E, Pastor-Gaitan P, Gutierrez-Perez JL. Impact of treatment on quality of life for oral and oropharyngeal carcinoma. Int J Oral Maxillofac Surg. 2009;38(10):1052–8. 19596557 10.1016/j.ijom.2009.06.008

[14] Kjeldsted E, Dalton SO, Frederiksen K, Andersen E, Nielsen AL, Stafström M, et al. Association between human papillomavirus status and health-related quality of life in oropharyngeal and oral cavity cancer survivors. Oral Oncol. 2020;109:104918. 32795908 10.1016/j.oraloncology.2020.104918

[15] Korsten LHA, Jansen F, Lissenberg-Witte BI, Vergeer M, Brakenhoff RH, Leemans CR, et al. The course of health-related quality of life from diagnosis to two years follow-up in patients with oropharyngeal cancer: does HPV status matter? Support Care Cancer. 2021;29(8):4473–83. 33454834 10.1007/s00520-020-05932-w PMC8236449

[16] McDowell L, Casswell G, Bressel M, Drosdowsky A, Rischin D, Coleman A, et al. Symptom burden, quality of life, functioning and emotional distress in survivors of human papillomavirus associated oropharyngeal cancer: an Australian cohort. Oral Oncol. 2021;122:105560. 34653749 10.1016/j.oraloncology.2021.105560

[17] Nemade H, Thaduri A, Gondi JT, Chava S, Kumar A, Raj P, et al. Five-year long-term functional and quality of life outcomes in total glossectomy survivors. Eur Arch Otorhinolaryngol. 2025;282(4):2063–70. 39511055 10.1007/s00405-024-09059-0

[18] Nuñez-Vera V, Garcia-Perla-Garcia A, Gonzalez-Cardero E, Esteban F, Infante-Cossio P. Impact of treatment on quality of life in oropharyngeal cancer survivors: a 3-year prospective study. Cancers (Basel). 2024. 10.3390/cancers16152724. 39123452 10.3390/cancers16152724 PMC11311390

[19] Plonowska-Hirschfeld KA, Gulati A, Stephens EM, Ochoa E, Xu MJ, Ha PK, et al. Treatment modality impact on Patient-Reported quality of life in human papilloma Virus-Associated oropharyngeal carcinoma. Laryngoscope. 2024;134(4):1687–95. 37767815 10.1002/lary.31065

[20] Pourel N, Peiffert D, Lartigau E, Desandes E, Luporsi E, Conroy T. Quality of life in long-term survivors of oropharynx carcinoma. Int J Radiat Oncol Biol Phys. 2002;54(3):742–51. 12377326 10.1016/s0360-3016(02)02959-0

[21] Ryzek DF, Mantsopoulos K, Künzel J, Grundtner P, Zenk J, Iro H, et al. Early stage oropharyngeal carcinomas: comparing quality of life for different treatment modalities. BioMed Res Int. 2014;2014:421964. 24719863 10.1155/2014/421964 PMC3955642

[22] Shivangi Sundram AA, Sourabh, Nandi, Sunil Saini. Assessment of oncosurgical and functional outcomes in patients undergoing glossectomy for advanced carcinoma tongue: A Cross-sectional study at a tertiary cancer care centre in Northern India. J Clin Diagn Res. 2024;18(2):5–12.

[23] Xu MJ, Plonowska KA, Gurman ZR, Humphrey AK, Ha PK, Wang SJ, et al. Treatment modality impact on quality of life for human papillomavirus-associated oropharynx cancer. Laryngoscope. 2020;130(2):E48-56. 30919470 10.1002/lary.27937

[24] Yin X, Shan C, Wang J, Zhang H. Factors associated with the quality of life for hospitalized patients with HPV-associated oropharyngeal squamous cell carcinoma. Oral Oncol. 2020;103:104590. 32050152 10.1016/j.oraloncology.2020.104590

[25] Yoshimura R, Shibuya H, Miura M, Watanabe H, Ayukawa F, Hayashi K, et al. Quality of life of oral cancer patients after low-dose-rate interstitial brachytherapy. Int J Radiat Oncol Biol Phys. 2009;73(3):772–8. 18676096 10.1016/j.ijrobp.2008.05.001

[26] Hjermstad MJ, Bergenmar M, Bjordal K, Fisher SE, Hofmeister D, Montel S, et al. International field testing of the psychometric properties of an EORTC quality of life module for oral health: the EORTC QLQ-OH15. Support Care Cancer. 2016;24(9):3915–24. 27113466 10.1007/s00520-016-3216-0

[27] Kosgallana S, Jayasekara P, Abeysinghe P, Lalloo R. Oral health related quality of life of oral cancer patients treated with radiotherapy alone or with chemotherapy in a tertiary referral centre in Sri Lanka. BMC Oral Health. 2023;23(1):162. 36935513 10.1186/s12903-023-02854-x PMC10024835

[28] Peters MDJ, Marnie C, Tricco AC, Pollock D, Munn Z, Alexander L, et al. Updated methodological guidance for the conduct of scoping reviews. JBI Evid Synth. 2020;18(10):2119–26. 33038124 10.11124/JBIES-20-00167

[29] Tricco AC, Lillie E, Zarin W, O’Brien KK, Colquhoun H, Levac D, et al. PRISMA extension for scoping reviews (PRISMA-ScR): checklist and explanation. Ann Intern Med. 2018;169(7):467–73. 30178033 10.7326/M18-0850

[30] Baumann I, Seibolt M, Zalaman I, Dietz K, Maassen M, Plinkert P. Quality of life in patients with oropharyngeal carcinoma after primary surgery and postoperative irradiation. J Otolaryngol. 2006;35(5):332–7. 17049151 10.2310/7070.2006.0077

[31] Jansen F, Verdonck-de Leeuw IM, Cuijpers P, Leemans CR, Waterboer T, Pawlita M, et al. Depressive symptoms in relation to overall survival in people with head and neck cancer: A longitudinal cohort study. Psychooncology. 2018;27(9):2245–56. 29927013 10.1002/pon.4816 PMC6231089

[32] In‘t Veld M, Jager DHJ, Chhangur CN, Ziesemer KA, Leusink FKJ, Schulten E, editors. Oral-functioning questionnaires in patients with head and neck cancer: a scoping review. J Clin Med. 2023;12(12). 10.3390/jcm12123964 PMC10299551 37373657

[33] Gomes E, Aranha AMF, Borges AH, Volpato LER. Head and neck cancer patients’ quality of life: analysis of three instruments. J Dent (Shiraz). 2020;21(1):31–41. 32158782 10.30476/DENTJODS.2019.77677.0 PMC7036356

[34] Kosgallana S, Jayasekara P, Abeysinghe P, Hjermstad M, Lalloo R. Translation and validation of Sinhala version of modified EORTC QLQ-OH15 in oral cancer patients who receive radiotherapy with or without chemotherapy in Sri Lanka. BMC Oral Health. 2022;22(1):359. 35986341 10.1186/s12903-022-02392-y PMC9392238

