# Supplementary material for: Oral health-related quality of life in oral cancer patients assessed with EORTC instruments: a scoping review
Source: BMC Oral Health. 2025 Nov 29;25:1854. doi: 10.1186/s12903-025-07280-9 (PMC12665205; doi:10.1186/s12903-025-07280-9)
Supplement: Supplementary file 1 — Supplementary Material 1. [file 12903_2025_7280_MOESM1_ESM.docx]

**Supplementary Material**

**Search Strategy**

*Appendix A.1. PubMed (n = 248)*

*("Mouth Neoplasms"[MeSH Terms] OR "Tongue Neoplasms"[MeSH Terms] OR "salivary gland neoplasms"[MeSH Terms] OR "Lip Neoplasms"[MeSH Terms] OR "Hypopharyngeal Neoplasms"[MeSH Terms] OR "Tonsillar Neoplasms"[MeSH Terms] OR "Oropharyngeal Neoplasms"[MeSH Terms] OR "Pharyngeal Neoplasms"[MeSH Terms] OR "Oral cancer"[Title/Abstract] OR "Mouth cancer"[Title/Abstract] OR "tongue cancer"[Title/Abstract] OR "salivary gland cancer"[Title/Abstract] OR "Lip cancer"[Title/Abstract] OR "hypopharyngeal cancer"[Title/Abstract] OR "tonsillar cancer"[Title/Abstract] OR "oropharyngeal cancer"[Title/Abstract] OR "pharyngeal cancer"[Title/Abstract] OR "hypopharyngeal cancer"[Title/Abstract] OR "Mouth cancer"[Title/Abstract] OR "Oral Squamous Cell Carcinoma"[Title/Abstract]) AND ("Patient Reported Outcome Measures"[MeSH Terms] OR "Quality of Life"[MeSH Terms] OR "health related quality of life"[Title/Abstract] OR "Oral Health-Related Quality of Life"[Title/Abstract]) AND ("European Organization for Research and Treatment of Cancer"[Title/Abstract] OR "EORTC"[Title/Abstract] OR "EORTC QLQ"[Title/Abstract] OR "EORTC QLQ-H&N35"[Title/Abstract] OR "EORTC QLQ-H&N43"[Title/Abstract] OR "EORTC QLQ-C30"[Title/Abstract] OR "EORTC QLQ-OH17"[Title/Abstract] OR "EORTC QLQ-OH15"[Title/Abstract]) AND "english"[Language] AND 2000/01/01:2025/12/31[Date - Publication]*

*Appendix A.2. Embase (n = 525)*

*('mouth tumor'/exp OR 'tongue tumor'/exp OR 'salivary gland tumor'/exp OR 'lip tumor'/exp OR 'hypopharynx tumor'/exp OR 'tonsil tumor'/exp OR 'oropharynx tumor'/exp OR 'pharynx tumor'/exp OR 'oral cancer':ti,ab OR 'mouth cancer':ti,ab OR 'tongue cancer':ti,ab OR 'salivary gland cancer':ti,ab OR 'lip cancer':ti,ab OR 'tonsillar cancer':ti,ab OR 'oropharyngeal cancer':ti,ab OR 'pharyngeal cancer':ti,ab OR 'hypopharyngeal cancer':ti,ab OR 'oral squamous cell carcinoma':ti,ab OR 'tongue squamous cell carcinoma':ti,ab) AND ('patient-reported outcome'/exp OR 'quality of life'/exp OR 'health related quality of life':ti,ab OR 'oral health-related quality of life':ti,ab) AND ('european organization for research and treatment of cancer':ti,ab OR 'eortc':ti,ab OR 'eortc qlq':ti,ab OR 'eortc qlq-h&n35':ti,ab OR 'eortc qlq-h&n43':ti,ab OR 'eortc qlq-c30':ti,ab OR 'eortc qlq-oh17':ti,ab OR 'eortc qlq-oh15':ti,ab) AND [english]/lim AND [2000-2025]/py*

*Appendix A.3.* Web of Science *(n = 272)*

TS = ("Mouth Neoplasms" OR "Tongue Neoplasms" OR "Salivary gland neoplasms" OR "Lip Neoplasms" OR "Hypopharyngeal Neoplasms" OR "Tonsillar Neoplasms" OR "Oropharyngeal Neoplasms" OR "Pharyngeal Neoplasms" OR “mouth tumor” OR “mouth tumor” OR “tongue tumor” OR “salivary gland tumor” OR “lip tumor” OR “hypopharynx tumor” OR “tonsil tumor” OR “oropharynx tumor” OR “pharynx tumor” OR “Oral cancer" OR "Mouth cancer" OR "tongue cancer" OR "salivary gland cancer" OR "Lip cancer" OR "hypopharyngeal cancer" OR "tonsillar cancer” OR "oropharyngeal cancer" OR "pharyngeal cancer" OR "hypopharyngeal cancer" OR "Oral Squamous Cell Carcinoma" OR "tongue squamous cell carcinoma") AND TS = ("Patient Reported Outcome Measures" OR "Quality of Life" OR "health related quality of life" OR "Oral Health-Related Quality of Life" OR “patient-reported outcome”) AND TS = ("European Organization for Research and Treatment of Cancer" OR "EORTC" OR "EORTC QLQ" OR "EORTC QLQ-H&N35" OR "EORTC QLQ-H&N43" OR "EORTC QLQ-C30" OR "EORTC QLQ-OH17" OR "EORTC QLQ-OH15") AND LA=(English) AND PY=(2000-2025)

*Appendix A.4.* CINAHL *(n = 65)*

((MH "Mouth Neoplasms”) OR (MH "Tongue Neoplasms") OR MH "Salivary Gland Neoplasms+") OR (MH "Lip Neoplasms") OR (MH "Hypopharyngeal Neoplasms" ) OR (MH "Tonsillar Neoplasms") OR (MH "Oropharyngeal Neoplasms+") OR (MH "Pharyngeal Neoplasms+") OR TI ("Oral cancer" OR "Mouth cancer” OR "tongue cancer" OR "salivary gland cancer" OR "Lip cancer" OR "hypopharyngeal cancer" OR "tonsillar cancer" OR "oropharyngeal cancer" OR "pharyngeal cancer” OR "hypopharyngeal cancer" OR "Oral Squamous Cell Carcinoma” OR "tongue squamous cell carcinoma”) OR AB ("Oral cancer" OR "Mouth cancer” OR "tongue cancer" OR "salivary gland cancer" OR "Lip cancer" OR "hypopharyngeal cancer" OR "tonsillar cancer" OR "oropharyngeal cancer" OR "pharyngeal cancer” OR "hypopharyngeal cancer" OR "Oral Squamous Cell Carcinoma” OR "tongue squamous cell carcinoma”)) AND ((MH "Patient-Reported Outcomes+") OR (MH "Quality of Life+") OR TI ("health related quality of life" OR "Oral Health-Related Quality of Life") OR AB ("health related quality of life" OR "Oral Health-Related Quality of Life")) AND ((TI ("European Organization for Research and Treatment of Cancer” OR "EORTC” OR "EORTC QLQ" OR "EORTC QLQ-H&N35" OR "EORTC QLQ-H&N43" OR "EORTC QLQ-C30" OR "EORTC QLQ-OH17" OR "EORTC QLQ-OH15") OR AB ("European Organization for Research and Treatment of Cancer” OR "EORTC” OR "EORTC QLQ" OR "EORTC QLQ-H&N35" OR "EORTC QLQ-H&N43" OR "EORTC QLQ-C30" OR "EORTC QLQ-OH17" OR "EORTC QLQ-OH15")) AND (LA English) AND (DT 20000101-20251231)

Search conducted on **March 4, 2025**.
